# Lignin from Brewers’ Spent Grain: Structural and Thermal Evaluations

**DOI:** 10.3390/polym15102346

**Published:** 2023-05-17

**Authors:** Oluwashina Philips Gbenebor, Oludolapo Akanni Olanrewaju, Mohammed Awwalu Usman, Samson Oluropo Adeosun

**Affiliations:** 1 Department of Metallurgical and Materials Engineering, University of Lagos, Lagos 101017, Nigeria; sadeosun@unilag.edu.ng; 2 Department of Industrial Engineering, Durban University of Technology, Durban 4000, South Africa; oludolapoo@dut.ac.za; 3 Department of Chemical and Petroleum Engineering, University of Lagos, Lagos 101017, Nigeria; musman@unilag.edu.ng

**Keywords:** cellulose, hemicellulose, lignin, lignocellulose, spent grain

## Abstract

Lignocellulose is a renewable ubiquitous material that comprises cellulose, hemicellulose, and lignin. Lignin has been isolated from different lignocellulosic biomass via chemical treatments, but there has been little or no investigation carried out on the processing of lignin from brewers’ spent grain (BSG) to the best of authors’ knowledge. This material makes up 85% of the brewery industry’s byproducts. Its high moisture content hastens its deterioration, which has posed a huge challenge to its preservation and transportation; this eventually causes environmental pollution. One of the methods of solving this environmental menace is the extraction of lignin as a precursor for carbon fiber production from this waste. This study considers the viability of sourcing lignin from BSG with the use of acid solutions at 100 °C. Structural and thermal analyses were carried out on extracted samples, and the results were compared with other biomass-soured lignin to assess the proficiency of this isolation technique. Wet BSG sourced from Nigeria Breweries (NB), Lagos, was washed and sun-dried for 7 days. Tetraoxosulphate (VI) (H_2_SO_4_), hydrochloric (HCl), and acetic acid, each of 10 M, were individually reacted with dried BSG at 100 °C for 3 h and designated as H2, HC, and AC lignin. The residue (lignin) was washed and dried for analysis. Wavenumber shift values from Fourier transform infrared spectroscopy (FTIR) show that intra- and intermolecular OH interactions in H2 lignin are the strongest and possess the highest magnitude of hydrogen-bond enthalpy (5.73 kCal/mol). The thermogravimetric analysis (TGA) results show that a higher lignin yield can be achieved when it is isolated from BSG, as 82.9, 79.3, and 70.2% were realized for H2, HC, and AC lignin. The highest size of ordered domains (0.0299 nm) displayed by H2 lignin from X-ray diffraction (XRD) informs that it has the greatest potential of forming nanofibers via electrospinning. The enthalpy of reaction values of 133.3, 126.6, and 114.1 J/g recorded for H2, HC, and AC lignin, respectively, from differential scanning calorimetry (DSC) results affirm that H2 lignin is the most thermally stable with the highest glass transition temperature (T_g_ = 107 °C).

## 1. Introduction

**Figure 1:**
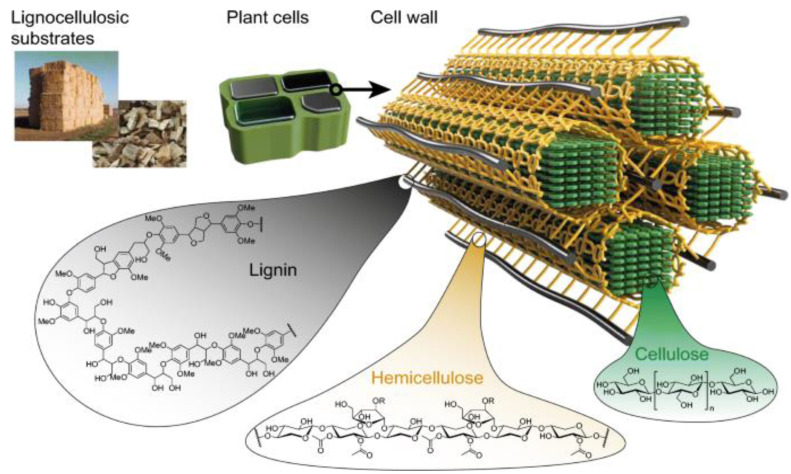
Cellulose, hemicellulose, and lignin in the plant cell wall [4].

Lignocellulose is the cheapest renewable material on earth, as its sources include products of agricultural wastes (biomass). The biomass comprises cellulose, hemicellulose, and lignin (Figure 1). Findings of Saha [1] indicated that lignocellulosic biomass comprises 35–50% cellulose, 20–35 hemicellulose, and 10–25% lignin. Rice straw, for instance, consists of 32–47% cellulose, 19–27% hemicellulose, and 5–24% lignin [2], where the cellulose and hemicellulose are closely packed by layers of lignin. Mansor et al. [3] noted that pineapple leaves contain 30% cellulose, 37% hemicellulose, and 22% lignin; its stem is made up of 46% cellulose, 29% hemicellulose, and 17% lignin, while 42, 32, and 17% of cellulose, hemicellulose, and lignin, respectively, are contained in pineapple root. This shows that the major component in lignocellulose is cellulose, followed by hemicellulose and lignin.

Cellulose is a linear homopolymer and the main component in the plant cell wall, which is responsible for its strength. It is the most abundant biopolymer on the planet, and it maintains the highest portion among the three biopolymers (cellulose, hemicellulose, and lignin) found in plants [5]. Hemicellulose is a heterogeneous polymer comprising pentoses, hexoses, glucose, and sugar acids. It is the second most common polysaccharide after cellulose [1]. Lignin is a three-dimensional, highly cross-linked macromolecule composed of three types of substituted phenols, which include coniferyl, sinapyl, and p-coumaryl alcohols. Lignin’s deposition in the cell wall is of great importance for plant development as it provides rigidity and strength to the cell wall, giving mechanical support to the plant organs. Acting as a glue that connects cellulose and hemicellulose, lignin presents hydrophobicity, thereby enabling the transport of water and solutes in the vascular system and protecting the cell against pathogens [6]. The type and distribution of lignin may vary in the same plant (including plants of different species). This implies that the content of lignin in the leaves may differ from that in the roots; the content in the stalks may differ from that present in the branches, etc. This, in turn, may propitiate having lignin of dissimilar properties from the same plant coupled with different extraction techniques being employed. Some of the areas of lignin application include concrete (for building construction), antioxidant, carbon fiber, plastics, paper, fuel, and UV filters [7,8,9]. Capecchi et al. [10] sourced lignin nanoparticles from fishery wastes for UV shielding, antioxidants, and antimicrobial bio-fillers. Lignin nanoparticles have been useful in the synthesis of bio-catalytic esters and lipophilic esters of hydroxytyrosol [11,12]. Ecofriendly electrochemical biosensors have been synthesized from kraft lignin nanoparticles [13]. Organosolv lignin has been affirmed to enhance the permeability features of in situ soils [14].

To effectively obtain one of these polymers from lignocellulose for economic use, chemical reagent(s) is/are used to break the structure of the unwanted polymers to isolate the desired one. Sodium hypochlorite (NaOCl) has been used to separate lignin, hemicellulose, and cellulose chemically from powdered sugarcane bagasse [15,16,17]. The biomass powder was bleached to leave the residue as cellulose and hemicellulose while dissolved lignin was contained in the filtrate. Further treatment with sodium hydroxide (NaOH) on the residue dissolved hemicellulose into the filtrate while pure cellulose was left as a residue. Potassium hydroxide (KOH) has been used on pulverized maize stalk to isolate cellulose (which turned out as residue) from hemicellulose and lignin; the latter constituents both dissolved in the solution as filtrate [18]. In the work of Abdul-Rahman et al. [19], NaOH was made to react with tea leaf waste. Lignin and hemicellulose were separated into the solution as filtrate, leaving cellulose as residue. Lignin precipitate was obtained from corn residues via agitated reaction with ethanol for 1 h at 60 °C [20]. To achieve lignin yield, three volumes of water were added to the filtrate at room temperature, and the pH was adjusted to 1. Lignin residue has been processed from banana skin when treated with 14 M and 20 mL tetraoxosulphate (VI) acid; H_2_SO_4_ [21] and dissolved cellulose formed in the filtrate. Formic acid/acetic acid solution in the mixing ratio 70:30 *v*/*v* was added to rice straw powder for lignin extraction [22]. After 180 min of reaction (at 90 °C), lignin (residue) was made to precipitate out of the mixture via centrifugation. Taleb et al. [23] employed the Klason procedure to extract lignin from spent coffee grounds with the use of 72% H_2_SO_4_ as the first step of hydrolysis. A gram of the biomass was mixed with 15 mL of the acid solution and stirred at 30 °C for 2 h. The second hydrolysis stage entailed adding 500 mL of deionized water to the mixture for further refluxing at 120 °C for 4 h. The resulting residue from the solution was termed “sulphuric acid lignin”. Previous studies have confirmed the successful isolation of lignin from numerous kinds of biomass. There has been, however, little or no investigation carried out on the processing of lignin from brewers’ spent grain, BSG, to the best of the authors’ knowledge. This material is a waste generated from the brewery industry and makes up 85% of its byproducts [24]. A major use of BSG is animal feed because of its high contents of fats, proteins, and minerals [25]; it has also been used as food additives and a source of amino acids and sugar [26,27]. The high moisture content in BSG expedites its deterioration, which has posed a huge challenge to its preservation and transportation [28]. The color and offensive smell of its deteriorated form is a problem to the environment and can be an issue in its utilization as food supplements. Karlsen and Skov [29] informed that BSG contains anti-nutritional agents such as lignin that limits the digestive activities in fish. The study reported the need to isolate lignin from BSG for a better use as aquafeeds. This research focuses on the feasibility of isolating lignin from BSG with the use of acid solutions. To assess the efficacy of the extraction process, lignin’s structural and thermal features are evaluated and compared with other biomass-soured lignin.

## 2. Materials and Methods

### 2.1. Isolation of Lignin from BSG

The wet BSG used for this study was sourced from Nigeria Breweries (NB), located in Lagos state, Nigeria. The BSG waste was thoroughly washed with distilled water and sun-dried for 7 days to prevent reactions that may engender further deterioration. As employed by Ajani et al. [21], 200 mL (30% *v*/*v*) of diethyl ether (99% purity) was added to every 100 g of dried BSG that was to be analyzed to remove extractives. After 24 h of soaking, the residue was washed with distilled water to neural pH and oven-dried at 80 °C for 4 h. To isolate lignin from the cellulose and hemicellulose present in the treated biomass, reactions involving 10 M of H_2_SO_4_ (98% purity), HCl (34% purity), and acetic acid (99.6% purity) were separately involved. Each BSG/acid solution was heated to 100 °C for 3 h with continuous stirring. The residue (lignin) was thoroughly washed with distilled water to neutralize the pH and dried in the oven at the same temperature and time as earlier. To be certain that cellulose and hemicellulose were dissolved by each reagent into the filtrate, 5 M NaOH (99% purity) was added to the filtrate to yield a precipitate which was affirmed to be cellulose; this implied that the filtrate left in the reaction would be hemicellulose (attention was not given to these two polymers, as they were not the focus of this investigation). In this study, lignin isolated using H_2_SO_4_ is designated as H2 lignin, while HC and AC lignin represent lignin processed from HCl and acetic acid, respectively.

### 2.2. Lignin Characterization

#### 2.2.1. Fourier Transform Infrared Spectroscopy

Functional groups present in the isolated lignin samples including BSG were noted with a Nicolet 6700 M device at Ahmadu Bello University, Kaduna, Nigeria. Little quantity (10 mg) from each particle was dispersed in KBr, and at a resolution of 4 cm^−1^, transmittance measurement was taken between 500–4000 cm^−1^.

#### 2.2.2. Thermogravimetric Analysis (TGA)

A TGA Q500 instrument was used to determine the cellulose, hemicellulose, and lignin contents in BSG. It was also used in determining the efficacy of isolation in H2, HC, and AC lignin. Two milligrams of each sample particle were heated to 750 °C at a 10 °C/min heating rate. Temperatures at the commencement (T_onset_) and end (T_finish_) of decomposition were determined from the TGA curves. The temperature at which the decomposition rate was maximum (T_max_) was measured from the derivative thermogravimetric (DTG) plots. The equipment is located at Ahmadu Bello University, Kaduna, Nigeria.

#### 2.2.3. X-ray Diffraction (XRD)

PANanalytical (Malvern, UK) Empyrean equipment, domiciled in the National Geosciences Research Laboratories Kaduna, Nigeria, was used. A monochromatic Cu Kα radiation (k = 1.5406), operating at 40 kV and 40 mA, was employed for this operation. The average crystallite size of lignin samples was calculated by using Scherrer’s equation as adopted by Li et al. [30] in Equation (1):

(1) D=kλ/ βcos θ

D is the average crystallite size; K is a constant (a dimensionless shape factor usually taken as 0.9); $\lambda \dot{(A})$ represents the wavelength of X-rays (1.5406$\;\dot{A}$); β (rad) is the width of the crystalline peak at half height; and θ (degree) is the diffraction angle.

#### 2.2.4. Differential Scanning Calorimetry (DSC)

A Mettler Toledo (Columbus, OH, USA) DSC equipment situated at Ahmadu Bello University, Kaduna, Nigeria, was used to determine the glass transition temperatures (T_g_) of isolated lignin. Samples were heated to 150 °C and the enthalpy change value (ΔH) for each thermal event was calculated by integrating the area of the curve under consideration using the expression shown in Equation (2).

(2) ΔH=∫Cp dT

Cp is the specific heat capacity (J/g); T is the temperature (°C). The Cp was obtained from the expression shown in Equation (3).

Cp = Heat flow/heating rate
(3)

A 10 °C/min heating rate was employed for this characterization.

## 3. Results and Discussion

### 3.1. Identification of Functional Groups and Hydrogen-Bond Interactions

**Figure 2:**
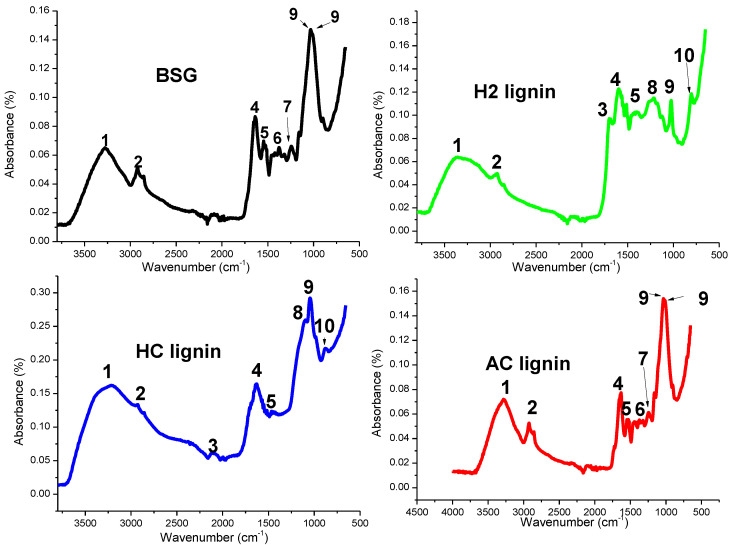
Functional groups in BSG and lignin samples.

The FTIR spectra of BSG and the lignin samples extracted with the different acid solutions are shown in Figure 2. Bands between 3800–3000 cm^−1^ show different areas of OH (in hydroxyl groups and phenolic compounds) occupancy, with H2 lignin existing on 3367 cm^−1^ while HC lignin was on 3349 cm^−1^. The spectra for BSG and AC lignin looked similar (but the latter showed a wider area) as the OH groups were formed on 3292 and 3269 cm^−1^. There were also spectra differences in the fingerprint regions for the samples between 1800–900 cm^−1^, as illustrated in Figure 2 and Table 1. Unconjugated C=O stretching was formed on 1700 cm^−1^ for both H2 and HC lignin samples but absent in BSG and AC lignin. Bending of CH_2_, found in cellulose and hemicellulose [31], was found on 1370 cm^−1^ in BSG and AC, showing that the cellulose and hemicellulose present in the BSG were not completely removed with the use of acetic acid during the extraction process. Lignin samples isolated using HCl and H_2_SO_4_ were devoid of these two polysaccharides, showing their effectiveness in the extraction process. Bands at 1226 cm^−1^ on the BSG and AC lignin represented the characteristic spectra of the syringyl ring in lignin [32], while this was represented at 1228 cm^−1^ in HC lignin and absent in H2 lignin. Aromatic C-H deformation in the syringyl ring was absent in the BSG and AC lignin but present on 1112 and 1109 cm^−1^ for the HC and H2 lignin samples, respectively. Aromatic rings in lignin were formed on 804 and 860 cm^−1^, which were not evident in BSG and AC lignin.

**Figure 3:**
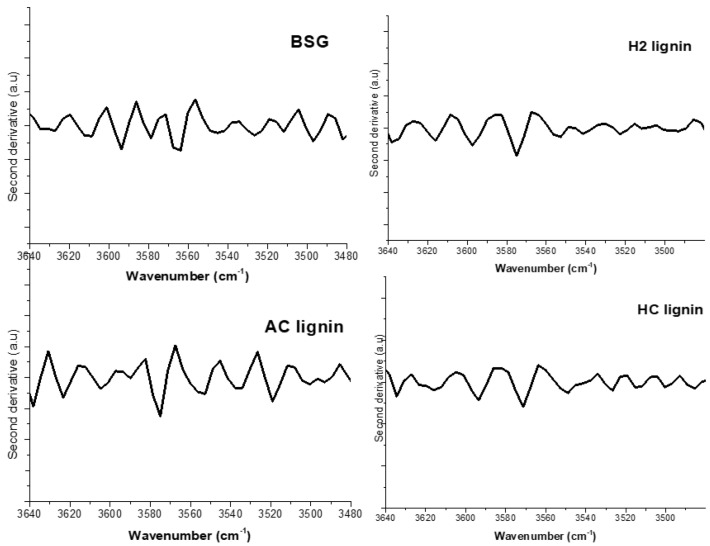
Second FTIR-derivative spectra of BSG, H2, AC, and HC lignin.

Intra- and intermolecular hydrogen bonds have been confirmed to exist in lignin with the OH groups and phenolic compounds (3640–3520 cm^−1^) as observed by Kubo and Kadla [32]. Poletto and Zattera [33] suggested that a preferable identification of these bands could be easily achieved by a second derivative of the OH group region for the samples, as shown in Figure 3.

According to Kubo and Kadla [32], free OH groups in lignin have two bands occurring between 3640–3616 cm^−1^. Bands representing intra- and intermolecular hydrogen bonds in the lignin phenolic group form between 3560–3550 cm^−1^ and 3520–3480 cm^−1^. In this study, the second derivative spectra illustrated in Figure 3 shows that the free OH groups in the phenolic compounds in H2 lignin were represented at 3634 and 3614 cm^−1^. The two bands demonstrated by this group also occurred on 3637 and 3616 cm^−1^ in HC lignin while bands 3637 and 3622 cm^−1^ were formed on AC lignin. Brewers’ spent grain had its free OH groups existing on 3635 and 3610 cm^−1^. Two bands at 3570 and 3549 cm^−1^ represented intramolecular hydrogen bonds in phenolic groups in H2 lignin while that in HC lignin was observed on 3574 and 3542 cm^−1^. An intramolecular bond in AC lignin was formed on 3574 and 3536 cm^−1^; spectra at 3579 and 3545 cm^−1^ represented the intramolecular bond in BSG. The presentation of bands 3526, 3521, 3519, and 3526 cm^−1^ also contributed to the presence of intermolecular bonds in the phenolic groups of H2, HC, AC lignin and BSG, respectively. The average strength of intra- and intermolecular interactions in lignin in this study was evaluated by calculating the wavenumber shift, Δυ_OH_ between hydrogen-bonded species, and free OH vibration stretching, as adopted by Poletto and Zattera [33]. The results (Table 2) show that for each wavenumber in the second derivative spectra, H2 lignin had the highest Δυ_OH_ value, followed by HC and AC lignin. This implies that the intra- and intermolecular OH interactions had the strongest effect in H2 lignin compared to the other extracted lignin samples. Lignin with this nature is expected to exhibit the highest resistance to structural distortion at room and elevated temperatures. More energy will be expended in breaking the hydrogen bonds in H2 lignin than in HC lignin, while the least magnitude of energy will be required in breaking the lignin in BSG since it possessed the least Δυ_OH_. The presence of cellulose, hemicellulose, and other extractives in BSG may be responsible for this.

The values of Δυ_OH_ were used in calculating the hydrogen-bond enthalpy of lignin samples using Equation (4) [33], while the hydrogen-bond distance *R* for the two bands assigned to phenolic groups in lignin was evaluated using Equation (5), as adopted by Pimentel and Sederholm [34].

(4) $$-\Delta H\;(\mathrm{kCal}/\mathrm{mol})=0.016\;\Delta \upsilon_{\mathrm{OH}}+0.63\;$$

ΔV = 4430 (2.84 − *R*) (5)

where ΔV = V_o_ − V. The value of V_o_ was taken as 3600 cm^−1^ (the monomeric stretching frequency); V represented the phenolic group frequencies (there were two of them) in the second derivative FTIR spectra.

Among the isolated lignin, the magnitude of the hydrogen-bond enthalpy in this study was observed to maintain the highest value in H2 lignin and the lowest in AC lignin (Table 3). The shortest bond length was recorded for H2 lignin (for each band category). In agreement with the findings of Poletto and Zattera [33], the strongest interaction existed between OH…H (donor and acceptor) atoms in H2 lignin, as the distance between them was the shortest. The wavenumber shift, hydrogen-bond enthalpy, and bond distance were therefore used in this study to justify why the intra- and intermolecular interactions in H2 lignin were the strongest. The hydrogen-bond properties of embedded lignin in BSG at each phenolic group band were very low compared to the isolated ones. The existence of volatile constituents, extractives, and other organic compounds other than lignin in the biomass could be responsible for this.

### 3.2. Thermal Profiles of BSG and Lignin Samples

**Figure 4:**
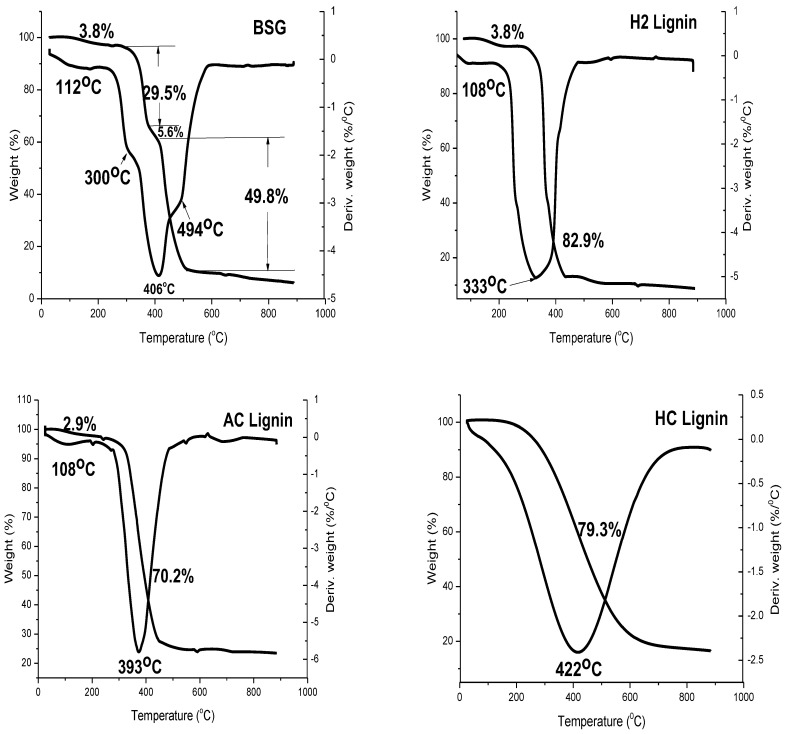
Thermal decomposition profiles of BSG, H2, AC, and HC lignin.

The thermogravimetric analysis result of BSG shown in Figure 4 had four decomposition stages. The first stage, which lay between 106–220 °C, represented the decomposition of moisture and volatile constituents within the sample. From this study, the content of matter within this temperature change was found to be 3.8%. Beyond this temperature, the decomposition of hemicellulose in BSG was exemplified by the second stage of decomposition. The thermal event commenced from 220–378 °C with 29.5% of the overall biomass (BSG) decomposed. The T_max_ from the DTG curve was 300 °C. The presence of hemicellulose in bagasse has previously been revealed by TGA/DTG analysis, where a peak temperature of 316 °C from the DTG result showed the appearance of hemicellulose [35]. Pyrolysis of biomass, as investigated by Yang et al. [36] and Chen et al. [37] via TGA, affirmed that hemicellulose degrades between 200–250 °C. The third stage of decomposition in the TGA/DTG curve showed the least biomass degradation of the three polysaccharides with 5.6% of the overall BSG decomposition. This biopolymer was observed to be lignin with its T_onset_, T_max_, and T_finish_ as 378, 406, and 420 °C, respectively. Lignin has been investigated to decompose over a wide range of temperatures based on the different thermal stabilities of numerous oxygen functional groups prevalent in its structure [38] and source. Lignin from soft wood *Pinus taeda* and hard wood *Eucalyptus grandis* decomposed with the T_max_ at 438 and 389 °C, respectively [38]. The polymer in BSG with the maximum content was cellulose, maintaining 49.8% of the BSG’s overall mass. Decomposition of BSG cellulose in this study commenced at 420 °C and finished at 513 °C while it attained a T_max_ of 494 °C. The TGA/DTG result for BSG obtained in this study implied that the biomass comprised hemicellulose, lignin (lowest content), and cellulose (highest content).

The isolation of lignin from BSG via chemical treatment justified the elimination of cellulose and hemicellulose, as shown in Figure 4. Two significant decomposition stages, which included moisture elimination and lignin decomposition, were observed for H2, AC, and HC lignin samples. According to Reodregues et al. [39], the thermal disintegration of the polymer structure in lignin began between 200–275 °C, while the major decomposition process started around 400 °C. This was associated with the formation of aromatic hydrocarbons, phenolics, hydroxyphenolics, and guaiacyl–syringyl-type compounds, with most products having phenolic OH groups. Complete lignin isolation with the use of H_2_SO_4_ (Figure 4) culminated in a higher yield compared to the untreated BSG as 82.9% decomposition of the overall mass attributed to lignin occurred. The decomposition commenced at 291 °C and stopped at 425 °C, with its T_max_ at 333 °C, which was higher than that of the lignin in BSG (300 °C). This implied that the complete removal of cellulose and hemicellulose improved the thermal stability of lignin. A major decomposition in the TGA of peanut shells and rice husks was observed to occur between 110–400 °C, which was the second stage of the pyrolysis process [40]; the decomposition was reported to be a result of chemically bound water, cellulose, hemicellulose, and lignin. In the works of Alzagameem et al. [41], the major structural decomposition of kraft lignin from black liquor took place between 260 and 478 °C, where cleavage of aryl ether links occurred due to its low thermal stability.

Two thermal events were also observed with the use of AC lignin (Figure 4). The first event entailed the decomposition of 2.9% (between 95 and 200 °C) moisture content and the release of volatile constituents such as carbon monoxide (CO) and carbon (IV) oxide (CO_2_). Oxidation of the aliphatic portions in the molecule as a result of an increase in the oxygen and a decrease in the hydrogen contents could have occurred within this temperature range [42]. Costa et al. [43] observed that a second-stage decomposition of sugarcane bagasse lignin occurred between 300–480 °C, indicating an increase in carbon content with increased liberation of volatiles like water vapor, CO, CO_2_, methanol (CH_3_OH), and methane (CH_4_). A lignin content of 70.2% was observed at the second decomposition event in AC lignin where the T_onse_t and T_finish_ were 283 and 447 °C, respectively. The creation of vinyl guaiacol, ethyl, and methyl byproducts is usually observed between 230 and 260 °C with the degradation of the propanoid side chains of lignin [44,45]. Alzagameem et al. [41] reported that condensation processes leading to the formation of unsaturated C=C bonds are affirmed to take place within 160–270 °C. A first-stage decomposition of a kraft water-soluble lignin microsphere occurred between 230–260 °C and was ascribed to the formation of low-molecular-weight products due to propanoid side chain cleavage [46]. The researchers confirmed that the main decomposition step occurred at elevated temperatures (250–450 °C), leading to the production of a large quantity of CH_4_ due to the cleavage of the main lignin chain. This was followed by several rearrangements and condensation reactions of the aromatic structure that led to the formation of char structures with its decomposition above 650 °C. The quantity of lignin extracted from the BSG using HCl was obtained as decomposition of 79.3% by mass took place (Figure 4). The thermal degradation of HC lignin started at 203 °C and ended at 628 °C. The comparison of the TGA/DTG results obtained in this study with earlier works has shown that lignin was successfully isolated from BSG.

### 3.3. Crystallographic Structure of BSG and Its Lignin Samples

**Figure 5:**
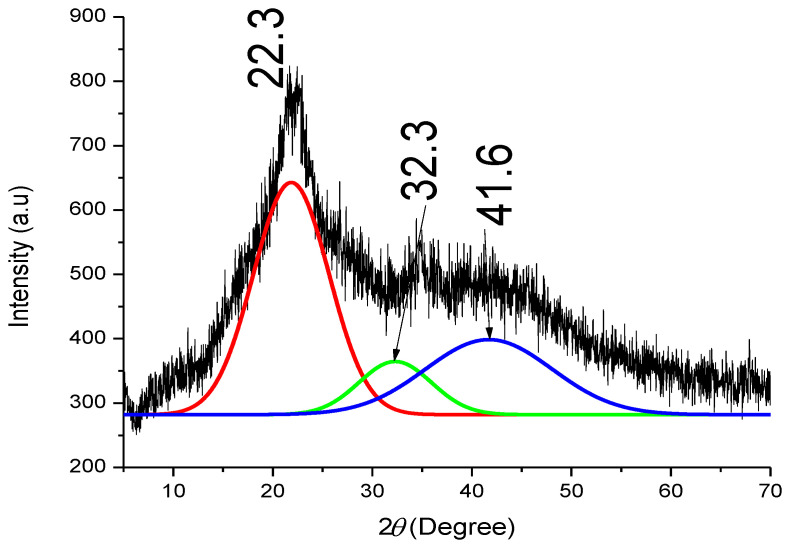
X-ray diffraction pattern of BSG.

The XRD pattern shown in Figure 5 indicates that BSG can also be a source of cellulose, hemicellulose, and lignin. As is typical of many lignocelluloses, the crystalline phase at (002) on 2θ = 22.3° represented the existence of cellulose, while the broad diffraction between 2θ = 30–55° showed the presence of amorphous constituents of lignin and hemicellulose. Patterns of rice wheat straw revealed that the biomass was composed of cellulose in its structure with the existence of disordered structures of lignin and hemicellulose [47]. The presence of weak and broad peaks in the diffraction pattern revealed these amorphous constituents. Particles of Albizia lebbeck Benth pods have also been observed to display a diffraction pattern similar to that obtained in this study [48]. The diffraction peaks in this broad region were resolved using the Gaussian method of peak deconvolution as conducted in earlier studies [49,50] with the maximum F number >10,000, corresponding to an R^2^ value of 0.99. A peak at 2θ = 32.3° representing a (040) crystallographic plane was formed in BSG with an indication that a cellulose phase exists within the broad region. Gbenebor et al. [50] observed a minor (broad and wide) cellulose peak sourced from bamboo particles at 2θ = 34.7°, while Mission et al. [51] confirmed that amorphous cellulose could exist between 2θ = 32 and 34°. This was further substantiated by Zhang et al. [52], where the presence of crystalline and amorphous cellulose was reported in corn leaves. The result obtained in this study thus showed that the amorphous cellulose type was formed in the broad diffraction peak, while the broader peak (41.6°) represented the presence of hemicellulose and lignin in BSG.

**Figure 6:**
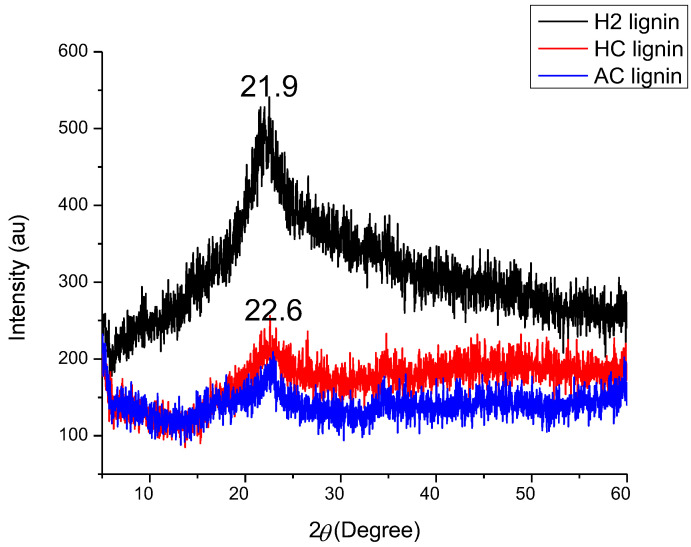
X-ray diffraction patterns of lignin samples.

Figure 6 shows the X-ray patterns of H2, HC, and AC lignin. All lignin samples showed a similar diffraction pattern, with the strongest occurring at 2θ = 21.9° for H2 lignin; the maximum peaks for HC and AC lignin were both similar at 2θ = 22.6°. The X-ray pattern of hard wood lignin as reported by Guodarzi et al. [53] was found at 2θ = 21.2°, while 2θ = 22.7° was characterized by lignin sourced from acetic acid-treated *Betula platyphylla* [32]. Lignin from black kraft liquor had its maximum peak diffracted on 2θ = 20.26°, while lignin macroparticles from the same source diffracted on 2θ = 22.94° [54]. The mean sizes of the ordered domains (crystallite) in each lignin structure as calculated from Scherrer’s equation (Equation (1)) were 0.0299, 0.0249, and 0.0226 nm for H2, HC, and AC lignin, respectively. Lignin extracted using H_2_SO_4_ on BSG possessed the highest size of ordered domains and hence had a high potential of forming nanofibers via the electrospinning of the three [55]. The reduced crystallite sizes of HC and AC lignin implied that their polydispersity indices were high (AC being the highest of the three) as each lignin comprised varying molecule sizes in its structure [53]. Non-uniformity in the sizes of molecules, according to Guodarzi et al. [53], may cause difficulty in fiber formation if HC and AC were electrospun; a portion of the lignin molecular weight would be spinnable while the other portion would encounter spraying of the solution.

### 3.4. Glass Transition Temperatures and Reaction Enthalpies of Lignin Samples

**Figure 7:**
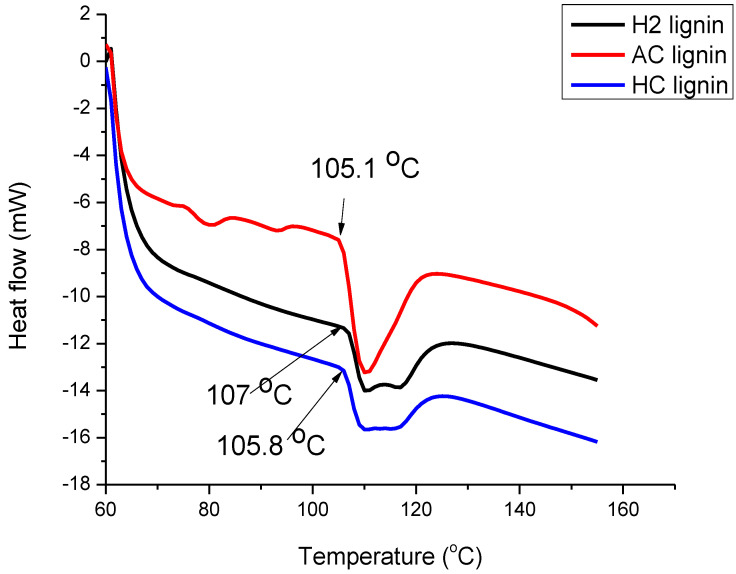
DSC profiles of H2, HC, and AC lignin.

The DSC curves for H2, AC, and HC lignin samples in Figure 7 show exothermic curves between 105 and 125 °C. The T_g_ of HC and AC lignin were 105.8 and 105.1 °C, respectively, which were lower than that of H2 lignin, maintained at 107 °C. This supports the claims of Feldman et al. [56] that the Tg of lignin lay between 100 and 180 °C. According to Buranov et al. [57], the existence of hydrogen bonding between phenolic OH groups coupled with an increased concentration of aromatic rings in the lignin chain was responsible for its high T_g_. This suggested that the H2 lignin in this study was characterized by effective hydrogen bonding; this was justified by it having the strongest strength offered by its intra- and intermolecular interactions, as measured by its wavenumber shift (see Table 2). The enthalpy of the reaction as calculated from the DSC curves revealed that the highest magnitude was obtained in the H2 lignin (133.2 J/g), while 126.6 and 114.1 J/g were measured for HC and AC lignin, respectively. This implied that H2 lignin was the most thermally stable of the three and that the energy required to break its lignin bond would be the greatest. Watkins et al. [58] reported that lignin of this form could serve as a flame retardant.

## 4. Conclusions

This study has proven that the largest byproduct from the brewery industry can be a good source of material important in the chemical, construction, and medical industries aside from its use as animal feed. Lignin, for the first time, has been successfully isolated from BSG using 10 M each of H_2_SO_4_, (98% purity), HCl (34% purity), and acetic acid (99.6% purity) solutions. The functional groups in BSG also existed in acetic acid lignin, making the spectra comparable with different intensities, indicating the prevalence of chemical reactions between acetic acid and BSG. Treatment with H_2_SO_4_ and HCl culminated in the additional existence of aromatic C-H deformation in the syringyl ring and aromatic ring formations. The wavenumber shift, bond enthalpy, and bond distance determined from FTIR were used in deciphering the intra- and intermolecular hydrogen-bond interactions within lignin’s structure. Extracting lignin from BSG using H_2_SO_4_ (H2 lignin) was the most efficacious of the three reagents; the lignin possessed the largest wavenumber shift, bond enthalpy, and lowest bond length. This implied that the bonds in H2 lignin would require the greatest magnitude of energy to be broken of the three lignin samples. Lignin extracted from BSG using H_2_SO_4_ was the most thermally stable, exhibiting a T_g_ of 107 °C, followed by HCl lignin with 105.8 °C, and the least stable was acetic acid lignin (105.1 °C). Thermogravimetry analysis showed that the BSG used in this study contained 5.6% lignin. Treatments with the acid reagents engendered enhanced lignin yields of 82.9, 79.3, and 70.2% as realized using H_2_SO_4_, HCl, and acetic acid, respectively. The highest size of ordered domains (0.0299 nm) exhibited by H2 lignin will enhance its ease of being processed into nanofibers via electrospinning. In summary, BSG is a good source of lignin, and a superlative yield can be achieved using H_2_SO_4_. The polymer can be used to improve the mechanical and thermal strength of other polymers when used as a reinforcement owing to its strong intra- and intermolecular OH interactions. Lignin processed from this biomass can be a precursor for carbon fiber production.

## Figures and Tables

**Table 1:** Summary of functional groups in BSG, AC, HC, and H2 lignin.

Functional Groups		Wavenumber (cm^−1^)		
	BSG	AC Lignin	HC Lignin	H2 Lignin
1. Free OH groups in alcoholic compounds	3292	3269	3349	3367
2. CH stretching	2923	2932	2916	2920
3. Unconjugated C=O stretching	--	--	1700	1700
4. Lignin aromatic ring stretching	1515	1515	1513	1580
5. Asymmetric CH deformation	1465	1465	1467	1470
6. CH_2_ bending in cellulose and hemicellulose	1370	1370	--	--
7. Syringyl ring in lignin	1226	1226	--	--
8. Aromatic C-H deformation in the syringyl ring	--	--	1112	1109
9. Aromatic ring and primary alcohol	1039, 1003	1039, 1003	1026	1041
10. Aromatic ring	--	--	804	860

**Table 2:** Wavenumber shifts of BSG and extracted lignin samples from second derivative of FTIR spectra.

	Sample Wavenumber (cm^−1^)				$\Delta \upsilon_{\mathrm{OH}}\;({\mathrm{cm}}^{-1})$		
BSG	H2	HC	AC	BSG	H2	HC	AC
3635	3634	3637	3637	343	386	366	358
3610	3614	3616	3622	318	363	345	343
3579	3570	3574	3574	287	317	303	295
3545	3549	3542	3536	253	298	271	257
3526	3526	3521	3519	234	275	250	240

**Table 3:** Hydrogen-bond enthalpy and bond distance of lignin samples.

Lignin Samples	Bands Assigned to Phenolic Groups (cm^−1^)	−ΔH (kCal/mol)	R(Á)
H2	3570	5.73	2.882
	3549	5.34	2.876
HC	3574	5.48	2.884
	3542	4.97	2.876
AC	3574	5.35	2.884
	3536	4.74	2.877
BSG	3579	5.22	2.885
	3563	4.68	2.878
